# Improved protein contact predictions with the MetaPSICOV2 server in CASP12

**DOI:** 10.1002/prot.25379

**Published:** 2017-09-29

**Authors:** Daniel W A Buchan, David T Jones

**Affiliations:** ^1^ Department of Computer Science University College London London UK

**Keywords:** contact prediction, machine learning, meta‐prediction, bioinformatics

## Abstract

In this paper, we present the results for the MetaPSICOV2 contact prediction server in the CASP12 community experiment (http://predictioncenter.org). Over the 35 assessed Free Modelling target domains the MetaPSICOV2 server achieved a mean precision of 43.27%, a substantial increase relative to the server's performance in the CASP11 experiment. In the following paper, we discuss improvements to the MetaPSICOV2 server, covering both changes to the neural network and attempts to integrate contact predictions on a domain basis into the prediction pipeline. We also discuss some limitations in the CASP12 assessment which may have overestimated the performance of our method.

## 1 INTRODUCTION

Sequence covariation analysis has emerged as a powerful technique for accurately predicting contacts in protein 3D structures (Marks et al. 2011, Jones et al. 2012, 2015; Kaján et al. 2014, Ma et al. 2014, Seemayer et al. 2014, Buchan and Jones 2017). These methods have now been shown to substantially outperform previous non‐covariation methods based on neural networks or Support Vector Machines (Taylor et al. 2014). Methods integrating covariation analysis demonstrated significant improvements in the contact prediction category in CASP11, where the best performing group (CONSIP2/MetaPSICOV) had a mean precision of 27% (over the top L/5 long‐range contacts) (Kinch et al. 2016). This was a marked improvement from the prior CASP10 where the best precision remained around 20% (Taylor et al. 2014).

For CASP12, we have continued to improve the CONSIP2 server we developed for CASP11 (Kosciolek and Jones 2016). Our new method, MetaPSICOV2 (entered in to CASP12 under the name ‘MetaPSICOV’ with group number 13), is based on the previously published MetaPSICOV method to derive covariation‐based contacts (Jones et al. 2015). At its core MetaPSICOV is a meta‐predictor based on different covariation prediction algorithms, including mfDCA (Kaján et al. 2014), CCMpred (Seemayer et al. 2014) and PSICOV (Jones et al. 2012). When there isn't sufficient sequence data available to allow effective covariation analysis, the neural network is able to exploit information from additional machine learning‐based methods to enable effective contact prediction across a range of scenarios.

In this article, we describe the performance of the MetaPSICOV2 server in the CASP12 experiment, highlighting examples which worked well and discussing areas where there could be further improvements. In the Materials and Methods section, we cover the improvements we've made, which follow on from our analysis of our prior performance in CASP11.

## 2 MATERIALS and METHODS

### 2.1 Method overview

The MetaPSICOV2 method follows the same broad prediction protocol as our prior CONSIP2 method. We outline this below and we refer interested readers to the earlier CONSIP2 article for more complete details (Kosciolek and Jones 2016). We also summarise below the significant differences made to the MetaPSICOV2 server entered in CASP12.

**Figure 1:**
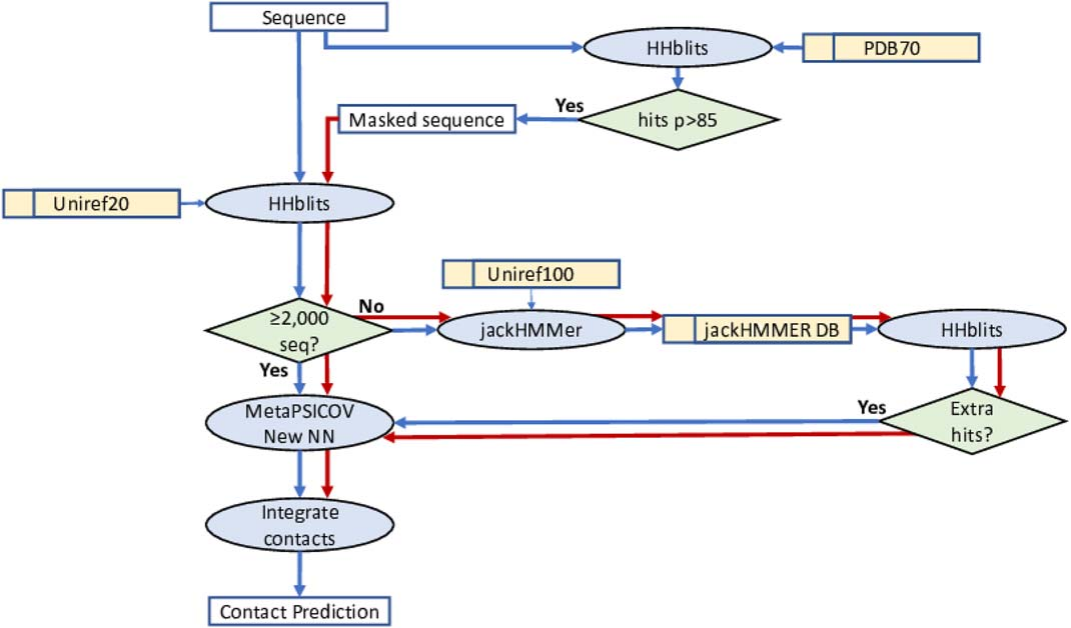
MetaPSICOV2 contact prediction pipeline. Sequences enter the pipeline at the top left. An HHblits run against PDB70 is run and if putative structural domains are identified, an additional masked sequence(s) is produced. The masked sequence (red path) and query sequence (blue path) then follow the CONSIP2 pipeline. If the prediction over the masked sequence produces high quality contacts these are integrated before the final Contact Prediction is produced

The core prediction pipeline remains as per the CONSIP2 method; the server begins by attempting to construct a large multiple alignment using HHblits by searching the Uniref20 sequence library (Remmert et al. 2011). When sufficient sequences are found (that is, >2 000) a MetaPSICOV contact prediction will proceed (Jones et al. 2015). When fewer than 2,000 sequences can be identified, we use jackHMMer (Eddy 1998) to search the Uniref100 sequence database. If any additional sequence relatives can be found these are used to compose an additional HHblits database. A further HHblits search of this new database can then build a new multiple sequence alignment. The MetaPSICOV2 server then utilises the largest alignment produced via either path for the MetaPSICOV prediction. Alongside this core pipeline we have added a number of changes, which are described below and summarised in Figure 1.

### 2.2 New neural network architecture

The MetaPSICOV2 neural network is an incremental development of the prior methodology. The principal change is a move to a slightly deeper and wider first‐stage network architecture composed of two hidden layers of 160 ReLU units, compared to a single hidden layer of 55 sigmoid units in the original method. Additionally, a wider input window of 15 residues is used, compared to 9‐residue window used in the MetaPSICOV method. Once again, the output layer is softmax, with a cross‐entropy loss function and SGD (stochastic gradient descent) training with momentum. The second‐stage filtering network remains unchanged from the original method, but now contributes far less to overall prediction accuracy, presumably because the additional hidden layer in the first stage is capable of performing much of the required filtering. The input features and training data set are unchanged from the original method.

In our own benchmarking on the original PSICOV test set of 150 large protein domain families, MetaPSICOV2 shows a modest improvement, giving a long‐range L precision of 53% compared to 51% for MetaPSICOV.

### 2.3 New domain splitting approach

For the CASP12 MetaPSICOV2 server, we implemented a simple approach to dealing with the issue of smaller Free Modelling (FM) domains being poorly predicted due to excessive alignment drift from large adjacent Template Based Modelling (TBM) domains. HHblits (Remmert et al. 2011) was used to search against the PDB70 HMM library with the complete target sequence. Local alignments to PDB70 with a match probability of ≥ 98% were then masked out as likely TBM regions. Any remaining unmasked regions of at least 30 residues were then rerun as separate domains and the new domain‐based contacts copied into the appropriate sections of the whole chain contact map (represented by the red path in Figure 1).

### 2.4 Number of effective sequences

Our contact prediction proceeds by first generating large sequence alignments. Typically, such large alignments will contain many redundant sequences. To get a better estimate of the true information content in each alignment, we calculate the Number of Effective Sequences, *N*_*eff*_ (Morcos et al. 2011, Skwark et al. 2014) with a clustering threshold of 62% sequence identity.

## 3 RESULTS

### 3.1 MetaPSICOV2 performance

Table 1 shows the performance of MetaPSICOV2 for the FM and FM/TBM CASP12 targets for the top L/5 predicted contacts. The mean precision over these 35 domains is 43.27% for the FM targets and 58.05% for the 13 FM/TBM targets. The median Number of Effective Sequences (*N_eff_*) is 42 and 289 for the FM and FM/TBM targets respectively. As the performance of MetaPSICOV is critically dependent on having large, diverse alignments the difference in performance between the FM and FM/TBM targets is easily explained by the increased *N_eff_* between the two categories.

**Table 1:** Summary of MetaPSICOV2 performance

Target ID	Domain	Precision (%)	*N_eff_*	Type
T0859	D1	4.35	1	FM
T0862	D1	26.32	9	FM
T0863	D1	12.82	80	FM
T0863	D2	6.94	80	FM
T0864	D1	64.00	175	FM
T0866	D1	100	952	FM
T0869	D1	52.38	16	FM
T0870	D1	8.00	28	FM
T0878	D1	43.48	204	FM
T0880	D2	25.00	1	FM
T0886	D1	100.00	1473	FM
T0886	D2	100.00	1473	FM
T0888	D1	4.00	2	FM
T0890	D2	13.64	16	FM
T0892	D2	63.64	289	FM
T0894	D1	0.00	16	FM
T0897	D1	3.57	10	FM
T0897	D2	16.00	10	FM
T0898	D1	27.27	33	FM
T0899	D1	86.54	109	FM
T0899	D2	61.11	109	FM
T0900	D1	95.24	7	FM
T0901	D2	50.00	631	FM
T0904	D1	25.49	42	FM
T0905	D1	93.88	914	FM
T0912	D3	42.86	1023	FM
T0914	D1	3.13	6	FM
T0914	D2	15.15	6	FM
T0915	D1	38.71	25	FM
T0918	D1	72.73	428	FM
T0918	D2	84.00	428	FM
T0918	D3	87.50	428	FM
T0923	D1	15.52	10	FM
T0941	D1	8.70	3	FM
T0946	D1	62.50	337	FM
T0868	D1	75.00	11	FM/TBM
T0884	D1	26.67	26	FM/TBM
T0890	D1	58.82	16	FM/TBM
T0892	D1	64.29	289	FM/TBM
T0894	D2	63.64	16	FM/TBM
T0896	D1	72.22	2673	FM/TBM
T0896	D2	10.00	3	FM/TBM
T0898	D2	27.27	33	FM/TBM
T0901	D1	80.00	631	FM/TBM
T0909	D1	24.62	80	FM/TBM
T0912	D2	88.24	1026	FM/TBM
T0943	D1	69.23	473	FM/TBM
T0945	D1	94.67	872	FM/TBM

Contact prediction precision is calculated over the top L/5 Long Range contacts. Where *L* is the length of the protein and Long Range is taken to be a sequence separation >23 residues.

*N_eff_* Gives the number of effective sequences calculated as described in the Materials and Methods.

Type gives the prediction category; FM: Free modelling, FM/TBM: Free modelling/Template Based Modelling.

**Figure 2:**
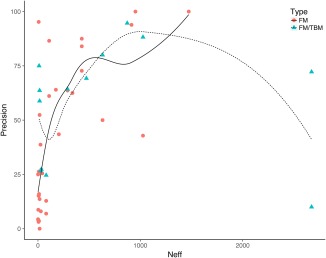
Figure shows the increase in precision as the *N_eff_* increases. FM targets are shown as red circles and FM/TBM targets as blue triangles. Trend lines shown have been fitted using LOESS

In Figure 2, we show the relationship between *N_eff_* and precision across the FM and FM/TBM targets. The general trend is that as *N*_eff_ increases, precision also increases for both the FM and FM/TBM targets. This reaches a maximum for the FM targets when *N*_eff_ approaches 1,500. In these cases, MetaPSICOV2 was able to achieve a precision of 100% for two targets, T0886‐D1 and T0886‐D2. Further to our previous work on EigenTHREADER (Buchan and Jones 2017), we note that with such high precision over the top L/5 contacts, it should be possible to uniquely specify the fold of the domain. For the FM/TBM targets precision also appears to increase with increasing *N_eff_*, although this appears to saturate and possibly tail off beyond *N_eff_* values of 1,500, although we are cautious of this interpretation given that there were relatively few examples of FM/TBM targets.

### 3.2 Notable predictions

In general, the best performing predictions are those with higher *N_eff_* values, and adequate predictive performance is achieved whenever *N_eff_* is >200.

Of particular note are domains T0886‐D1 and T0886‐D2, where MetaPSICOV2 achieved a precision of 100% over the top L/5 contacts. Visual inspection of the native structure indicates that T0886 is a structure with 2 discrete domains with little inter‐domain interaction in the structure. The two domains possess similar beta‐sandwich folds although the secondary structure connection topology is different. The generated multiple sequence alignments for this target show consistent, ungapped alignments over the 2 discrete domain regions with a short linker region which has several gaps, such that the alignment coverage over the domain regions is excellent.

The lowest precision (of zero) was seen on target domain T0894‐D1. Examining the alignment produced, there are no sequence relatives which are aligned to the entirety of the T0894 sequence. Sequences which align at either the C‐terminal or N‐terminal regions show very poor alignment to the other terminal region. In the region of the first domain, the aligned proteins are very sequence‐homogeneous with large blocks of absolute sequence conservation, and this is reflected in the low *N_eff_* value (16).

Interestingly, target T0900 has a very high precision (95.24) despite a very low *N_eff_* value (7). We note than many other CASP12 entrants, including many server groups, achieved a fairly high accuracy in both modeling and contact prediction for this target. The fold is a two sheet beta sandwich with substantial structural similarity to a number of carbohydrate binding domains with classic “jelly‐roll” folds. Our performance here likely reflects only that this target was somewhat “easy” for all groups, and that there were similar folds in the MetaPSICOV2 training set, which might well imply that it was not really an FM target.

In general, the best and worst performances of MetaPSICOV2 highlight the critical importance of both alignment size (in terms of *N_eff_*) and alignment quality when resolving accurate contact predictions. Future increases in the size of the sequence databases or improvements in the sensitivity of sequence searching methods will both be likely sources of increased performance for covariation‐based contact prediction.

### 3.3 Domain identification performance

In 10 cases, our new domain identification process produced an updated set of contacts, in comparison to running just the default MetaPSICOV2 pipeline pathway (see Table 2). Contacts generated via this domain recognition pathway are more precise in half of these cases. In the other cases, there is no change in the measured precision, indicating both that the added contacts were not in the top L/5 and, positively, that this additional branch in the pipeline does not degrade performance. The mean improvement in precision is 12.3%, although typically, the improvement is <3%. Targets T0946‐D1 and T0896‐D1 showed significant increases in precision of 56.25% and 61.11%, respectively.

**Table 2:** Change in performance for integrating domain predictions

Target ID	Default Precision (%)	Updated Precision (%)	*N_eff_*	Type
T0862	26.32	26.32	9	FM
T0904	25.49	25.49	42	FM
T0905	93.88	93.88	914	FM
T0941	7.25	8.70	3	FM
T0946‐D1	6.25	62.50	337	FM
T0896‐D1	11.11	72.22	2673	FM/TBM
T0896‐D2	10	10.00	3	FM/TBM
T0909	23.08	24.62	80	FM/TBM
T0912‐D2	88.24	88.24	1026	FM/TBM
T0945	92	94.67	872	FM/TBM

Change in precision for targets where the new domain identification strategy was utilised.

Default Precision shows the predicted precision given the default MetaPSICOV2 pipeline.

Updated Precision shows the precision after integrating contacts based on domain recognition.

Type gives the prediction category; FM: Free modelling, FM/TBM: Free modelling/Template Based Modelling.

For T0946‐D1, the global alignment built by the default MetaPSICOV2 prediction pathway is fragmentary with a large number of gaps and there are no well aligned regions along the length of the target sequence. However, identifying domain regions did allow the sequence searches to find many shorter close homologues over the initial, D1, domain region. With a compact and diverse alignment for this region the contact prediction was significantly better.

For T0896, the default alignment search process fails to build a deep alignment with a great number of sequence relatives over the whole sequence. The *N_eff_* for the global alignment is just 5, which explains the low precision (11.11%). Using a domain‐based sequence search, the first domain (D1) produces an alignment with a *N_eff_* of 2,673 over that region. Again, with this larger and more diverse alignment in hand a much higher quality contact prediction was achievable for the first domain with a precision of 72.22%.

### 3.4 Neural network assessment

The CASP12 assessment suggests there was a substantial increase in performance from the MetaPSICOV/CONSIP2 to MetaPSICOV2 algorithms between CASP11 and CASP12, representing an increase in precision approaching 20%. While we would of course welcome such an improvement, we also wished to assess the extent to which this improvement was due to the additional changes in the neural network algorithm or the makeup of the targets and available sequences.

**Figure 3:**
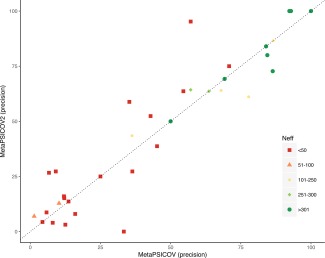
Comparison of precision values for top L/5 predictions using MeatPSICOV and MetaPSICOV2. Targets compared are only those domains which did not go through the MetaPSICOV2 domain recognition process. Points are individual CASP12 targets. Points are labelled by *N_eff_*: red squares and triangles for low *N_eff_* values, green diamonds and circles for high *N_eff_* values

To assess this, we calculated contact predictions using our earlier MetaPSICOV/CONSIP2 protocol for all the CASP12 targets where MetaPSICOV2 also did not attempt a domain‐based prediction. Comparing just these targets allows us to isolate improvements in the neural network architecture from those that came from the domain recognition process (covered above). Figure 3 shows the comparison in performance in terms of prediction precision over the top L/5 predictions and additionally labelled by *N_eff_*. Although not obvious, MetaPSICOV2 predictions represent an increase in performance of ∼1.8% (43.6% vs 45.5%), with 16 targets lying to the left of the diagonal, indicating improved MetaPSICOV2 performance, and only 10 lying to the right.

Labelled by *N_eff_*, the plot recapitulates the trends seen in Figure 2. As *N_eff_* increases so does precision (that is, moving from red squares toward green circles). Interestingly, we note that when *N_eff_* is below 100, MetaPSICOV2 is able to achieve precision values above 50% (5 cases) and MetaPSICOV is never equivalently performant for such very low‐*N_eff_* targets.

Notably, there is at least one outlying target, T0894‐D1, where MetaPSICOV2 fails to make any correct predictions, and so is substantially outperformed by the earlier MetaPSICOV. Omitting this outlier suggests that the average increase in precision for MetaPSICOV2 would be closer to 2.8%, which would be in line with our own prior neural network benchmarking.

This analysis suggests that the bulk of the increase in performance seen between CASP11 and CASP12 comes down to the CASP12 sequences being substantially easier prediction targets than those from CASP11, at least from a contact prediction perspective.

### 3.5 Contact probability estimates

**Figure 4:**
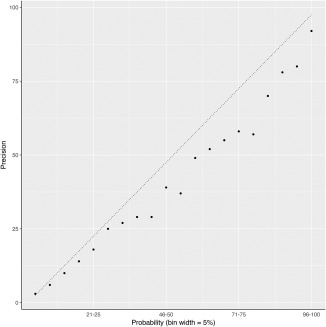
Relationship between MetaPSICOV2 probability estimate (in bins of 5%) and the true precision for predicted contacts which fell in those bins

We were interested to see how accurately MetaPSICOV2 could estimate the probabilities of predicted contacts. Obviously, a good contact prediction method should not only provide a low false positive rate, but should also accurately estimate the precision of predicted contacts. Figure 4 shows the relationship between MetaPSICOV2's probability (or precision) estimates for each predicted contact and the true precision based on benchmarking. The data shown is calculated over the complete list of contact predictions for all FM and FM/TBM targets.

The relationship is clearly not perfect, with the MetPSICOV2 probability values consistently overestimating (points lying to the right of the line) the true precision. This is likely a consequence of the CASP12 FM and FM/TBM targets being harder prediction targets than the MetaPSICOV2 training data. However, as the relationship is near to linear across the whole probability range, this suggests MetaPSICOV2 probability estimates are both reliable confidence indicators for any given contact prediction and provide a meaningful means to rank the predicted contacts.

## 4 DISCUSSION

The assessment of MetaPSICOV2 in CASP12 indicates that the server and underlying algorithm continue to be a strong and reliable predictor of protein contacts. However, our analysis suggests that the ease of the prediction targets this year is likely overinflating improvements in the method (and all other methods) since CASP11 by a considerable margin. Our assessment suggests that the new neural network architecture and domain recognition improvements in MetaPSICOV2 likely increase the predictive performance by no more than >5%, with 13% of the CASP assessed improvement purely a consequence of the makeup of the target set and changes to the number of available sequences since CASP11. A 5% gain is, of course, a considerable positive change but is substantially less than suggested by the overall changes observed between CASP11 and CASP12 by the assessors.

MetaPSICOV2 was able to build very large and diverse alignments (*N*_*eff*_ > 500) for at least six of the Free Modelling targets and these made a significant contribution to the MetaPSICOV2 performance in this year's experiment. We note that the median *N_eff_* remained similar between our CASP11 and CASP12 results (44 vs 42 respectively). In CASP11, we saw only one FM target with a *N_eff_* value >500. Omitting the six high *N_eff_* targets gives a precision of 35%, which is more in keeping with the improvement in performance we have estimated. In the future, when it is somewhat easy to find homologues, such targets might be better placed in one of the Template Based Modelling categories, at least in our opinion.

It is clear from Figure 3 that there remain some classes of target where MetaPSICOV/CONSIP2 still outperformed our updated MetaPSICOV2 pipeline. This indicates that there is still room to improve the training and neural network architecture of MetaPSICOV2 such that it will generalise better. The good performance on some very low‐*N_eff_* alignments also suggests the possibility of further improvements in training neural networks to better handle shallow alignments.

## SOFTWARE

MetaPSICOV2 is now available via http://bioinf.cs.ucl.ac.uk/downloads/MetaPSICOV
